# Hospital Fall Prevention: A Systematic Review of Implementation, Components, Adherence, and Effectiveness

**DOI:** 10.1111/jgs.12169

**Published:** 2013-03-25

**Authors:** Susanne Hempel, Sydne Newberry, Zhen Wang, Marika Booth, Roberta Shanman, Breanne Johnsen, Victoria Shier, Debra Saliba, William D Spector, David A Ganz

**Affiliations:** * RAND CorporationSanta Monica, California; † Veterans Affairs Greater Los Angeles Healthcare SystemLos Angeles, California; ‡ University of California at Los Angeles/JH Borun CenterLos Angeles, California; § Agency for Healthcare Research and QualityRockville, Maryland; ¶ David Geffen School of Medicine, University of California at Los AngelesLos Angeles, California

**Keywords:** fall prevention, implementation, hospital, systematic review

## Abstract

**Objectives:**

To systematically document the implementation, components, comparators, adherence, and effectiveness of published fall prevention approaches in U.S. acute care hospitals.

**Design:**

Systematic review. Studies were identified through existing reviews, searching five electronic databases, screening reference lists, and contacting topic experts for studies published through August 2011.

**Setting:**

U.S. acute care hospitals.

**Participants:**

Studies reporting in-hospital falls for intervention groups and concurrent (e.g., controlled trials) or historic comparators (e.g., before–after studies).

**Intervention:**

Fall prevention interventions.

**Measurements:**

Incidence rate ratios (IRR, ratio of fall rate postintervention or treatment group to the fall rate preintervention or control group) and ratings of study details.

**Results:**

Fifty-nine studies met inclusion criteria. Implementation strategies were sparsely documented (17% not at all) and included staff education, establishing committees, seeking leadership support, and occasionally continuous quality improvement techniques. Most interventions (81%) included multiple components (e.g., risk assessments (often not validated), visual risk alerts, patient education, care rounds, bed-exit alarms, and postfall evaluations). Fifty-four percent did not report on fall prevention measures applied in the comparison group, and 39% neither reported fidelity data nor described adherence strategies such as regular audits and feedback to ensure completion of care processes. Only 45% of concurrent and 15% of historic control studies reported sufficient data to compare fall rates. The pooled postintervention incidence rate ratio (IRR) was 0.77 (95% confidence interval = 0.52–1.12, *P* = .17; eight studies; *I*^2^: 94%). Meta-regressions showed no systematic association between implementation intensity, intervention complexity, comparator information, or adherence levels and IRR.

**Conclusion:**

Promising approaches exist, but better reporting of outcomes, implementation, adherence, intervention components, and comparison group information is necessary to establish evidence on how hospitals can successfully prevent falls.

In-hospital falls are a significant clinical, legal, and regulatory problem, but information on effective fall reduction is lacking. The Centers for Medicare and Medicaid Services no longer reimburses hospitals for in-hospital falls with trauma.^1^ As the U.S. population ages, fall prevention is more relevant than ever; older, frail individuals are more prone to falls, and the consequences of falls are more severe.^2^,^3^

Preventing falls in U.S. acute care hospitals poses particular challenges, given that patients are acutely ill and average only 4.9 days in the hospital.^4^ This compressed acuity places a greater burden on staff to keep patients safe, so results from fall prevention interventions in long-term care facilities may not apply to acute care settings. Similarly, results from the international literature, where hospital stays are longer, may not generalize to U.S. hospitals.

Fall prevention programs are typically complex, involving multiple components that depend on leadership involvement and the cooperation of frontline staff from multiple disciplines. Programs may require potent monitoring strategies to ensure that staff adhere to implemented care protocols. Recent reviews provide limited evidence for acute care settings.^3^,^5^–^7^ It was hypothesized that the confluence of an effective strategy to implement interventions into clinical practice in acute care settings, the intervention components chosen, the type of monitoring strategies used to ensure adherence, and the baseline level of care intensity provided in the comparison group would determine a fall prevention program's success.

A systematic review was performed documenting implementation strategies, intervention components and comparators, adherence information, and the effectiveness of published fall prevention approaches in U.S. acute care hospitals.

## Methods

Studies were identified through existing reviews and an update search for original studies. The Database of Abstracts of Reviews of Effects (DARE), the Cochrane Database of Systematic Reviews, PubMed (applying a systematic review search filter), and existing fall prevention toolkits or guidelines were searched to identify reviews. PubMed, *Cumulative Index to Nursing and Allied Health Literature* (CINAHL), and Web of Science from January 2005 were searched to August 2011 for studies that existing reviews had not yet captured.^3^,^5^,^6^,^8^,^9^ A combination of free text words and the Medical Subject Heading term “accidental falls” restricted to hospital settings and English-language publications was used. The search was not limited to a set of known interventions, so diverse approaches were identified; the strategy is documented in detail elsewhere.^10^ Additional studies were identified through reference mining of included studies and consultation with experts in hospital-based fall prevention. Separate searches were performed for psychometric properties of risk assessment scales applied in included studies.^10^

Two independent reviewers screened titles and abstracts and full text publications. One reviewer abstracted study details, and a second checked them. Two independent reviewers rated implementation strategy intensity, intervention complexity, comparator information, and adherence levels. A statistician extracted study outcomes. Discrepancies were resolved through team discussion. The review protocol has been registered in PROSPERO, an international register of systematic reviews (ID CRD42011001593).

### Inclusion Criteria

Studies had to meet the following criteria:

#### Participants

Studies evaluating fall reduction interventions in hospitalized individuals were eligible for inclusion. Studies to reduce falls among staff, community-dwelling individuals visiting the hospital for treatments (outpatients), or day-hospital patients were excluded.

#### Interventions

Eligible interventions had to be aimed at reducing falls in the hospital. Studies evaluating discharge planning interventions focusing on the time after the hospital stay and outpatient programs were excluded. Studies evaluating interventions to reduce restraints, the risk of injuries from falls, or the effect of falls were excluded unless combined with other interventions aiming to reduce falls.

#### Design

Studies that reported on patient falls in the hospital for an intervention group and a concurrent or historic comparator (randomized controlled trials, controlled clinical trials, cohort studies comparing two cohorts, before–after studies, time series) were eligible for inclusion in the review. Descriptions of interventions without data or without comparators and case studies of individual patients were excluded.

#### Outcome

Studies had to report on the outcome of inpatient falls. Only studies reporting numerical data on the intervention and a comparison group or data on reduction in the number of falls or rate of falls relative to the comparator were considered. Publications were excluded if they plotted fall events on graphs without reporting numerical data, reported only a range of reductions across departments without exact incidence data for intervention and control groups, reported only descriptive and nonnumerical assessments (“fall rates improved”), or reported only on falls after discharge and other long-term effects.

#### Setting

Eligible studies were restricted to acute care U.S. hospital settings. Studies aimed at nursing homes, residential care facilities, and other long-term nonhospital care facilities were excluded. Facilities that reported average lengths of stay of more than 30 days were deemed not to be acute care and were excluded.

### Data Abstraction and Analysis

Information on study design, setting, participant characteristics, implementation strategies, intervention target, intervention components and comparators, information on adherence to care processes, and study results were extracted, as were type of hospital and wards and the description of the participant sample. Information on all descriptions of how the intervention was introduced into clinical practice (implementation strategies) were extracted. The main target of the intervention (staff, equipment, patients) was classified, and information on all reported intervention components (care processes used in the intervention group), context information relevant to fall prevention (existing fall prevention measures also present in the control group or before the intervention), and which tools were used was extracted. Intervention components aimed at all patients were differentiated from care processes for individuals identified as being at high risk of falling. Information on strategies fostering adherence to the implemented care processes was documented, and data on the intervention fidelity was extracted.

To broadly categorize the included studies, the intensity of the implementation strategy, the complexity of the intervention, the information on fall prevention activities in the comparison group, and the level of adherence to the intervention were rated as low, medium, or high. The ratings and further details of the rating criteria are documented in Table S1. Intraclass correlations (ICCs) were used to estimate rater agreement.

Information on number of fallers, number of falls, fall rate (per 1,000 patient days), and number eligible to fall in both groups was extracted. To evaluate the fall rate, an incidence rate ratio (IRR) was estimated for each study. The IRR is the ratio of the postintervention (or treatment group) fall rate to the preintervention (or control group) fall rate. An IRR less than 1 indicates a lower postintervention (or treatment group) fall rate than the preintervention (or control group) rate. The number of falls was used to estimate the standard deviation of the IRR.

Studies were pooled in a random effects model estimating the IRR and 95% confidence interval (CI). Meta-regressions were used to investigate the effect of implementation, intervention complexity, comparator information, and adherence. Heterogeneity was assessed using the *I*^2^ statistic. Potential for publication bias was assessed using the Egger regression and the Begg rank test.

## Results

**Figure 1:**
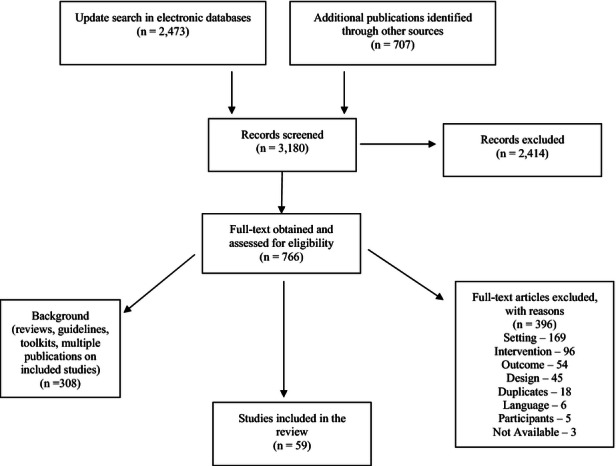
Flow diagram.

Figure 1 shows the study flow. The searches identified 3,180 publications. The electronic update search for fall prevention interventions retrieved 2,473 publications. The full text of 766 publications was obtained and screened against the inclusion criteria. Table S1 summarizes the details of the 59 studies meeting inclusion criteria. Interrater agreements (ICCs) were 0.87 (implementation intensity), 0.62 (intervention complexity), 0.69 (comparator information), and 0.75 (adherence levels).

### Study Characteristics

Studies were published over a period of 28 years. Studies with concurrent controls and historic controls were stratified. Four randomized controlled trials (RCTs) and seven nonrandomized studies reporting on an intervention and a concurrent control group were identified. Two RCTs were randomized at the patient level; two were cluster RCTs randomizing hospital wards or entire hospitals to the intervention or the control group. Forty-eight studies evaluated the success of interventions by comparing the number of falls or fall rates with those from a historic period before implementation of the intervention. The historic-control studies included a small number of time series reporting on three or more time points before and after the introduction of the intervention.

Studies varied in the reach of the intervention; 39 targeted selected hospital wards or units, 16 evaluated changes in an entire hospital, and four evaluated more than one hospital. Consequently, the number of included patients varied widely, ranging from fewer than 50 to more than 10,000 eligible participants per study.

### Implementation

Table 1 shows the employed implementation strategies (efforts to introduce the intervention into clinical practice), including staff education, establishing teams, piloting the intervention with input from front-line personnel to refine the intervention, leadership support, and continuous quality improvement procedures such as Plan-Do-Study-Act or the Institute for Healthcare Improvement Framework for Spread to promote unit-level buy-in. Ten studies (17%) reported no information on how the intervention was implemented. Twenty-nine studies (49%) reported primarily staff education, often to raise awareness of fall prevention or provide training for tools. Twenty studies (34%) described a comprehensive implementation strategy using continuous quality improvement models and a multifaceted strategy to integrate the intervention into clinical practice.

**Table 1:** Implementation, Intervention Components, Comparator, and Adherence in Included Studies

Implementation Strategies	Intervention Components for All Patients	Intervention Components for High-Risk Patients Only	Comparator Information	Adherence Strategies and Fidelity
Staff education to raise awareness of fall prevention or training for specific tools^3–7,12–15,17–19,21–32,34,35,38–45,47–49,51,53,55,57,58^	Fall risk assessment^2–4,6,13,14,16–21,23,26,29–34,36–40,43–45,47–51,53,55–59^	Alert signs placed on beds, doors, patients'' records^2–6,13,17–21,26,29,31,32,36,38,40,41,43–45,48,49,51–53,55,56,59^	Risk assessment^2,4,6,10,14,15,29,33,34,48,50,56^	Audit and feedback on adherence to processes of care^1–9,11,12,15,17,19,21,27,28,31,34,37,39,42–44,49,51,57^
Interdisciplinary team, task force or other hospital committee established^13,17,18,26,28,29,31,33,34,36,38,41,42,48,49,53,55,57,59^	Postfall evaluations^12,13,17,20–22,26,29,34,38,47,49,51,55–57^	Care, safety, and toileting rounds^6,7,12,13,16,18–21,24,29,30,34,38,40–43,45,47,48,52,53,55–57^	Restraints^10–12,24,31,48,51,54,58^	Monitoring and disseminating data on falls^5,6,12,14,19,28,32,42,43,53,56,57,59^
Piloting the intervention in selected units^2,26,29,31,36,39,44,45,47,49,53,55,59^	Patient and family education^4,6,26,33,34,45,47,49,53,55–57^	Bed- or chair-exit alarm systems^1,4,6,9,10,12,17,21–23,26,27,29,30,32,34,40,41,43,47,51,55,57,58^	Alert signs placed on beds, doors, patients'' records^2,4,6,15,32,56^	Fall prevention included in electronic health record^2,4,45–47,49^
Activities to raise leadership awareness or gain support^3,12,13,42,44,57,59^	Care, safety, and toileting rounds^7,16,21,38,41,52^	Patient and family education^2,13,14,17,19–21,29–32,36,41,43,44,48,50^	Other strategies^1,2,4,6,8–11,15,22,27,29,32,41,48–51,53,54,56,57^	Other adherence-promoting strategies^5,8,9,15–17,19,21,22,27,28,31,32,43,45,53,57,59^
Continuous quality improvement techniques; Plan-Do-Study-Act, Institute for Healthcare Improvement spread framework^2,28,42,49,52,55,57^	Awareness posters^5,26,33,56^	Identification wrist bands^3,6,17,21,26,29,34,41,44,47,49^	No information on existing fall prevention measures^3,5,7,13,16–21,23,25,26,28,30,35–40,42–47,52,55,59^	No specified adherence strategy and no fidelity data^10,13,14,18,20,23–26,29,30,33,35,36,38,40,41,48,50,52,54,55,58^
Other implementation strategies^8,14,17,27–29,34–36,47,49,53,57^	Clutter-free, safe environment efforts^6,45,50,53^	Bed side rails^1,4,20,38,43–45,48,50,54^		
No specified implementation strategy^1,9–11,16,20,37,46,50,54^	Medication review^14,16,33,35^	Low beds^1,4,27,29,34,43,44,48^		
	Low beds^45,50,53^	Nonskid socks and footwear^1,20,26,36,43,44,47,48^		
	Call lights within reach enforcement^34,53^	Use of sitters^21,40,50,53–56,59^		
	Nonskid socks and footwear^21,50^	Care plan communicated at change of shift report^5,13,17,18,38,49,51,55^		
	Other intervention components^4,8,15,17,19,26,27,33–35,41,42,45,47,50,52–57^	Moving high-risk patients close to nurses'' station or cluster^6,12,13,29,30,42,47,59^		
		Medication review^6,26,44,46,49,57^		
		Call lights within reach enforcement^4,7,20,43,48,50^		
		Clutter-free, safe environment efforts^18,26,38,44,50^		
		Bedside commode^1,29,43^		
		Other intervention components^1–5,10,12–15,17–22,25,26,29–34,37–42,44,45,47–51,53–57^		

References in this table are found in the online supporting information.

### Intervention Components

Table 1 shows intervention components documented in the literature, stratified according to care process aimed at all admitted patients and components applied only to patients classified as being at high risk for falls. Common components targeting all patients included fall risk assessment, patient and family education, and structured postfall evaluations. Commonly applied components for patients identified as being at at high risk of falling included alert signs placed on beds, doors, patient records, and call buttons in the nurses'' station; care, safety, and toileting rounds and ambulation assistance; bed-exit alarms; education; identification wrist bands or other markers; bed side rails; use of sitters; low beds; nonskid footwear; moving high-risk patients closer to the nurses'' station; communicating the care plan; medication review; and enforcing that call lights are within reach. Several studies used additional and less-common approaches, such as designating a specifically equipped fall prevention room on the ward.^11^

Most identified studies addressed fall prevention using multiple components but 11 of 59 (19%) described one-dimensional interventions such as the introduction of a bed-exit alarm with or without fall risk assessment. Twenty-six of all included studies (44%) were classified as intense interventions combining a large number of care processes with pertinent technology (e.g., bed-exit alarms, computerized decision support) or regular and resource-intense components such as staff providing scheduled toileting or the use of sitters to supervise patients continuously. The remaining studies (22/59, 37%) described a limited number of different intervention components.

Most interventions (48/59, 81%) targeted primarily healthcare provider behavior (e.g., introducing a new risk assessment or care protocol) rather than patients directly (e.g., through patient education) or equipment (e.g., introduction of a new bed-exit alarm). Several studies emphasized the introduction of a standardized care plan specifying universal and mandatory care processes triggered by a given risk score. Interventions were often aimed at improving the documentation and use of existing fall prevention measures rather than introducing new care processes.

#### Fall Risk Assessment

Forty-three (83%) studies incorporated patient-level fall risk assessment. This assessment determined which intervention components patients received. Table 2 shows the risk assessment tools together with published reliability and validity characteristics where available in the literature. The most commonly used published tool was the Morse Fall Scale (6/43 studies). More than half of studies (23/43) used a tool without known psychometric properties.

**Table 2:** Psychometric Properties of Risk Assessment Tools Used in Included Studies

Published Tool	Psychometric Performance Source	Tool Description	Acute Care Data^a^	U.S. Data^b^	Reliability Across Studies	Validity Across Studies
ADAPT Fall Assessment Tool^15^	Individual study^15^	ADAPT computerized information system, fall risk embedded into routine assessment documentation, allows customized interventions for specific patient risks, risk information integrated into care plan, report sheets, care conferences	Yes	Yes	n/a	Concurrent validity: risk assessment correlates 0.96 with Hendrich scale scores
Berryman Predisposition for Falling scale^44^ (applied to at-risk patients)	Review, data from^1^ study60	Assessed domains: age, mental status, length of stay, elimination, falling within the past 6 months, visual impairment, confined to chair, blood pressure	No	Yes	n/a	Face validity: most falls (3 VA patient care units observed for 3 months) were in patients aged ≥70
Hendrich, Hendrich II Fall Risk Model / Assessment^4,29,33,36,56^	Review, data from 1 study^61^	Assessed domains: mental state, gait and mobility, fall history, elimination, diagnosis, continence, mood, dizziness, weakness	Yes	n/a	n/a	Predictive validity: sensitivity 0.77, specificity 0.72
I'M SAFE Fall Risk Assessment Tool, Children's Hospital Denver^45^	Individual study^62^	Assessed domains: environment, history of falls, intravenous medications, orthopedic and muscular, rehabilitation and occupational and physical therapy, seizures andepilepsy	Yes	Yes	Internal consistency (α) 0.69	n/a
Innes Score; St Francis Memorial Hospital Standard Care Plan for the High-Risk Patient^31,32,48^	Systematic review, data from 1 study^63^	Assessed domains: previous trauma, disorientation, impaired judgment, sensory disorientation, muscle weakness, multiple diagnoses, language barrier	n/a	No	n/a	Predictive validity: sensitivity 0.89 (95% CI = 0.78–0.96), specificity 0.74 (95% CI = 0.72–0.75); PPV 0.07 (95% CI = 0.05–0.10), NPV 1.00 (95% CI = 0.99–1.00), OR = 23 (95% CI = 10.1–55.5)
Morse Falls Scale^1,2,6,16,28,39^	Systematic review, data from 4 studies^64^	Assessed domains: history of falling, presence of secondary diagnosis, use of ambulatory aids, administration of intravenous therapy, type of gait, mental status	Yes	n/a	n/a	Predictive validity: sensitivity 0.72–0.96, specificity 0.51–0.83
	Systematic review, data from 2 studies^63^	Score of 45 used as cutoff	Yes	No		Predictive validity: sensitivity 0.73–0.96, specificity 0.54–0.75, PPV 0.04–0.10, NPV 0.99–1.00.
	Systematic review, data from 3 studies^65^	6 items	Yes	n/a	Interrater agreement 0.96–0.98	Predictive validity: sensitivity 0.72–0.83, specificity 0.51–0.68
Schmid Fall Risk Assessment Tool^49,51^	Systematic review, data from 1 study^63^	Assessed domains: gait, confusion, assisted toileting, fall history, anticonvulsants; 5 items; score of 3 used as cutoff	n/a	Yes	n/a	Predictive validity: sensitivity 0.93 (95% CI = 0.80–0.98), specificity 0.78 (95% CI = 0.73–0.83), PPV 0.37 (95% CI = 0.27–0.47), NPV 0.99 (95% CI = 0.96–1.00), OR = 44.3 (95% CI = 13.2–172.4)
	Systematic review, data from 2 studies^65^	17 items; score 3 used as cutoff	Yes	Yes	Interrater agreement 0.88	Predictive validity: sensitivity 0.91–0.93, specificity 0.25–0.78
Timed Up & Go test^17^	Systematic review, data from 1 study^65^	Score 10–12 used as cutoff	No	n/a	Interrater agreement 0.56–0.99	Construct validity: judged as “good”
Unpublished tool, tool shown and risk factors reported^3,13,14,18,20,21,23,26,30,34,37,38,43,47,50,53–^^55,57,58^	n/a	n/a	n/a	n/a	n/a	n/a
Tool not described^19,40,59^	n/a	n/a	n/a	n/a	n/a	n/a
No risk assessment^5,7–12,22,24,25,27,35,41,42,46,52^	n/a	n/a	n/a	n/a	n/a	n/a

References in this table are found in the online supporting information.

a Tool tested in acute care setting.

b Applied in U.S. organization.

n/a = not available, not applicable; VA = Veterans Affairs; ADAPT = Assess: Disorientation, Activity, Postmedication, and Toileting.

### Comparator

Thirty studies (51%) did not report on existing, routine fall prevention measures already in place before the intervention or in a control group (the comparator of the study). Twenty-three studies (39%) reported some existing care processes such as risk assessment, whereas six (10%) were identified that already had an intense fall prevention program in place before the new intervention was established.

### Adherence Strategies and Fidelity Data

Table 1 shows the employed strategies aimed at facilitating adherence to the intervention components, care processes, and use of selected tools. These included audit and feedback of adherence to processes of care, monitoring and disseminating fall data, and integrating risk assessments into an electronic health record. Fidelity data (evidence of the uptake of intervention components) was reported in only 13 studies (22%). Twenty-three studies (39%) neither reported data nor described an adherence strategy.

### Outcomes and Effectiveness Results

The majority of authors reported positive changes (Table S1), although of 17 publications reporting a statistical test, only eight indicated significant improvement. Five of 11 studies with concurrent controls reported sufficient detail to calculate a fall rate, and the pooled intervention effect (IRR) was 0.92 (95% CI = 0.65–1.30; *P* = .64). There was evidence of heterogeneity across studies (*I*^2^: 68%). Seven of 48 studies with historic controls (15%) reported sufficient data to compare the fall rate before and after the intervention. Twenty studies neither reported the fall rate nor provided sufficient data to compute it; 21 studies reported the fall rate without the number of falls in both groups. The intervention effect across historical control studies (IRR) was 0.77 (95% CI = 0.50–1.18; *P* = .23; *I*^2^: 95%; seven studies).

Table 3 shows the fall prevention approaches for the 12 studies for which an IRR could be calculated. Five of the eight successful approaches (IRR < 1) described an implementation strategy such as staff education; combined a number of intervention components such as fall risk assessment, education, alert signs, and bed-exit alarms; and with one exception, audited adherence to the care processes,^12^–^16^ but other multifaceted approaches were not successful,^17^ there were other, less complex, successful approaches,^18^ and the number of reported strategies was not significantly different between studies.

**Table 3:** Evidence Table of Included Studies Reporting Fall Incidence Rate Ratios

		Implementation Strategies							Intervention Components															Comparator; Existing Strategies in Control Group			Adherence Strategies				Fall Rate		Log Scale Fall Incidence Rate Ratio (95% Confidence Interval)
									
Study	Setting	Staff Education	Team, Task Force	Pilot Intervention	Leadership Support	Continuous Quality Improvement, Spread Techniques	Other	None Specified	Fall Risk Assessment	Alert Signs	Education	Rounds	Bed-Exit Alarms	Postfall Evaluation	Bed Side Rails	Low Beds	Identification Band	Nonskid Footwear	Clutter-Free Environment	Medication Review	Sitters	High-Risk Near Nurses	Other Components	Risk Assessment	Other Strategies	No Information	Monitoring Data on Falls	Care Audit/ Feedback	Other Strategies	None Specified	Before or Control	After or Intervention	
Concurrent control																																	
Dykes, 12^2^	8 units in 4 urban hospitals			X		X			X	X	X												X	X	X			X	X		4.64	3.48^a^	0.75^a^ (0.55–1.02)
Hunderfund, 20^4^	Neurology unit and 6 medical units in tertiary care hospital	X							X	X	X		X		X	X							X	X	X			X			2.99 5.69	4.12	1.38 a (1.05–1.82) 0.72 (0.54–0.98)
Krauss, 13^6^	4 general medicine floors in urban, 1,300-bed tertiary care academic hospital	X							X	X	X	X	X			X	X		X	X		X	X	X	X		X	X			6.85	5.09 a	0.74 a (0.53–1.05)
Padula, 2011^8^	3 medical–surgical units in teaching hospital						X																X		X			X	X		2.80	3.20 a	1.14^a^(0.54–2.42)
Spetz, 2007^10^	Postneurosurgery unit in acute care hospital							X					X										X	X	X					X	6.12	2.79^a^	0.46^a^ (0.10–1.99)
Before-after study design																																	
Barker, 14^13^	2 psychiatric units in acute care hospital	X	X		X				X	X	X	X		X								X	X			X				X	6.84	5.10	0.75 (0.59–0.94)
Dacenko-Grawe, 15^21^	325-bed teaching hospital	X							X	X	X	X	X	X			X	X			X		X			X		X	X		4.04	2.77	0.69 (0.56–0.84)
Geffre, 19^23^	6 medical units (medical, oncology, surgical, telemetry, transitional care, rehabilitation)	X							X				X													X				X	2.04	1.52	0.75 (0.50. 1.12)
Lane, 21^37^	Medical–surgical and critical care units in metropolitan community hospital							X	X														X			X		X			2.27	3.89	1.71 (1.49–1.97)
Peterson, 18^46^	Medical, surgical, neurology, and gynecology services of urban 720-bed tertiary care hospital							X												X						X			X		6.40	2.80	0.44 (0.27–0.70)
Rainville, 17^48^	Medical surgical units in 248-bed facility	X	X						X	X	X	X			X	X		X					X	X	X					X	7.76	7.74	1.00 (0.58–1.71)
Weinberg, 16^57^	714-bed tertiary care teaching hospital	X	X		X	X	X		X		X	X	X	X						X			X		X		X	X	X		3.60	1.94	0.54 (0.43–0.68)

Note: References in this table are found in the online supporting information.

a Compared with a concurrent control group.

**Figure 2:**
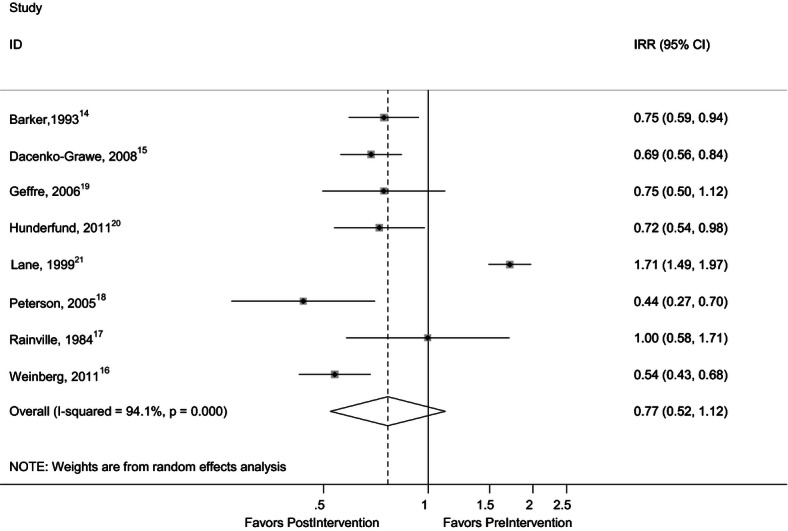
Log scale fall incidence rate ratio (IRR) status before and after the intervention. CI = confidence interval.

Across all studies that reported fall rates before and after the intervention (historic and concurrent control group studies), the pooled postintervention effect (IRR) was 0.77 (95% CI = 0.52–1.12; *P* = .17; eight studies^14^–^21^), as shown in Figure 2. There was evidence of substantial statistical heterogeneity (*I*^2^: 94%). Omitting each study in turn from the analysis showed a statistically significant postintervention effect when excluding one study^21^ (IRR = 0.67, 95% CI = 0.58–0.77) and a substantial reduction of heterogeneity (*I*^2^: 39%).

### Meta-Regressions and Publication Bias

Meta-regressions showed that, as the adherence level increased, the IRR decreased (*P* = .005) in studies with concurrent controls (five studies). This result indicates that larger intervention effects were observed in studies with greater evidence of adherence to intervention components, although the effect was not replicated in the analysis comparing pre- and postintervention data (*P* = .79, eight studies). None of the meta-regressions showed a statistically significant effect for implementation intensity, intervention complexity, or comparator information.

The quality of the reporting may have confounded results; excluding studies with little or no information and comparing only medium- and high-intensity studies showed a significant effect of intervention intensity (*P* < .001), although this result was based on six pre–post data studies only and was not replicated in the studies with concurrent controls (*P* = .70, four studies). The adherence effect was significant in the sensitivity analysis for concurrent controls (*P* = .001, four studies) but was not present in the pre–post data studies (*P* = .49, five studies).

In the few studies reporting analyzable data, no evidence of publication bias was identified (controlled trials: Egger test *P* = .75, Begg test *P* > .99, five studies; pre–post data analysis: Egger test *P* = .16, Begg test *P* = .71, eight studies).

## Discussion

The literature was systematically screened, and 59 U.S. acute care hospital studies reporting evaluations of fall prevention approaches were identified. Only a fraction reported sufficient data to compare fall rates, and pooled estimates found no statistically significant intervention effect. The implementation strategies were sparsely documented; most interventions included multiple components; information on the comparator was often absent; and many studies neither reported data on, nor described, adherence strategies to monitor completion of care processes.

Most interventions were unique approaches combining a number of different components and care processes aiming to prevent falls, such as risk assessment, visual alerts indicating risk, patient and family education, care rounds, bed-exit alarms, and postfall evaluations. Some components, such as screening patients for fall risk, were employed in almost all studies. A large number of interventions were applied only to patients identified as being at high risk. The overall success of such interventions may depend on the accuracy of the risk assessment in ensuring that the right patient is targeted. More than half of the included studies did not use published validated scales but instead developed their own tools, for which no psychometric data were reported. The sensitivity and specificity of even well-known tools are limited; the author of STRATIFY, one of the best-documented tools, concluded that it may not be optimal for identifying high-risk individuals for fall prevention.^22^

The large number of fall prevention studies identified that reported on U.S. acute care hospitals could provide great insight for clinicians and policy-makers on effective and less-effective strategies for reducing the risk of falls, but only a small proportion of studies reported sufficient data to evaluate the effectiveness of their approach, particularly among historical control studies. Assessing changes in the outcome of patient falls, a rare event that is subject to fluctuation, is challenging; to evaluate the effect of an intervention, the number of falls—together with the number of patients at risk or the fall rate—needs to be reported for similar study periods.^10^ One study summarizing a systematic review on fall prevention published in 1998 indicated that the usefulness of published evaluations is limited because of small sample sizes, the research design used, and study quality.[Bibr b7] The current study found the even more basic problem that data were not described sufficiently to enable effects to be evaluated.

In the few studies that reported data, the pooled intervention effect estimate was not statistically different from the preintervention status or standard care control group. Results of meta-analyses summarizing the international literature vary and report, for example, a statistically significant effect for historic control studies but not for controlled trials^23^ or no consistent results across outcomes (rate ratio vs number of fallers).^8^ Patient falls are not a novel problem in hospitals, so to understand the effect of a new intervention, the comparator status (part of the intervention context) needs to be known (which fall-reduction strategies were in place before the tested intervention or in a concurrent control group). The comparator is an important determinant of the success (the achieved change) of the intervention. Unfortunately, fewer than half of the included studies reported on existing, routine fall prevention approaches present in the comparator group. Recent publications have emphasized that, to comprehend study effects, more information is needed on the context in which interventions take place.^24^ Similarly, details of the implementation process have been singled out as a crucial element in patient safety practice evaluations to advance the science of patient safety,^25^ but information on how a fall prevention intervention was introduced into clinical practice in the target organization, for example through staff education or known continuous quality improvement strategies, was seldom documented.

Individual study results varied, and there was evidence of statistical heterogeneity between studies. It was hypothesized that the implementation intensity, intervention complexity, comparator information, and adherence to care processes were effect modifiers for the effectiveness of interventions to reduce falls, but the large majority of included studies could not be statistically analyzed. Meta-regressions showed some evidence of the importance of adherence levels (data on whether the intervention took place as intended and implemented care processes were indeed adhered to) and the intensity of the intervention, but effects were not consistent across available data. Adherence strategies are of particular importance for long-term changes. Initial success might not be maintained because adherence to introduced care processes fades in clinical practice, use of the introduced risk assessment tool may not be sustained, and recommended measures may no longer be systematically applied. Some barriers encountered in clinical practice included forgetting to remove identification signs next to call lights after high-risk patients were discharged and failing to educate new staff about fall prevention programs.^26^,^27^

This systematic review relied on published information. The amount of reported details, in particular regarding implementation and adherence strategies, may depend on a journal's word limit and preferences. Contacting primary authors may have provided answers to unresolved questions, but fall prevention interventions are only as good as their implementation and adherence strategies, and sufficient data to communicate the nature of the comparator and its intensity are crucial to understanding study effects. The Standards for QUality Improvement Reporting Excellence (SQUIRE) criteria provide detailed guidance for how complex interventions to improve the quality of healthcare delivery should be reported.^28^

Low statistical power limited these quantitative analyses. The absence of definitive findings should therefore not be interpreted as evidence that implementation strategies, intervention complexity, and level of adherence are unimportant. Until better data are available, readers may benefit from reviewing the successful studies documented in this review and pursuing approaches that are most compatible with their hospital culture and patient populations.

Promising approaches exist, but better reporting of outcomes and detailed information on intervention components and comparison groups, as well as the implementation strategy and adherence to care processes, need to be included in published fall prevention evaluations to establish a strong evidence base for successful interventions to reduce patient falls in hospitals.

## SUPPORTING INFORMATION

**Table S1.** Evidence Table.
