# Supplementary material for: Hospital Fall Prevention: A Systematic Review of Implementation, Components, Adherence, and Effectiveness
Source: J Am Geriatr Soc. 2013 Mar 25;61(4):483–94. doi: 10.1111/jgs.12169 (PMC3670303; doi:10.1111/jgs.12169)
Supplement: Supplementary file 1 [file jgs0061-0483-SD1.docx]

**Online Table 1: Evidence Table**

| **ID** | **Setting** | **Implementation (score)** | **All pts**  **vs Control** | **High risk**  **vs Control** | **IC** | **C** | **Adherence and fidelity data (score)** | **Falls results as reported** |
| --- | --- | --- | --- | --- | --- | --- | --- | --- |
| **Concurrent control** |  |  |  |  |  |  |  |  |
| Cozart (2009)[^1^](#_ENREF_1) RCT | A neurology and rehabilitation unit in a VA hospital | Pilot possible but setting and intervention components unclear (0) | n/a | 8 fall prevention rooms equipped with safety equipment (low beds, bed alarms, commode, non-skid socks and slippers, hipsters, suitable lightings, bed trapeze, side rails, non-skid shower mats, falls prevention poster)  vs 8 control rooms (non-skid socks, bracelets to signal fallers) | 1 | 1 | Daily room assessment by PI (Equipment Safety Checklist). Data n/a (1) | 1 fall in the intervention group vs 3 falls in the control group (p=0.306) |
| Dykes et al. (2010)[^2^](#_ENREF_2) Cluster RCT | 8 units in 4 urban US hospitals | IHI framework for spread to promote unit-level buy-in; tool kit developed in iterative process including end users (2) | Electronic risk assessment (Morse Fall Scale)  vs staff education, Morse Fall scale | Health information technology falls prevention tool kit (Fall T.I.P.S.) including risk assessment and printable posters; tailored bed posters above bed for pts at risk, includes status updates; pt education handouts for pt / family; plans of care automatically generated by tool kit; pt-specific alerts  vs usual care (generic ‘high risk for falls’ sign above bed, education handouts for pt / family; care plan manually in paper or electronic record) | 2 | 1 | Tool kit includes adherence dashboard to facilitate monitoring. 81% adherence to daily MFS completion in control, 94% in intervention units; toolkit outputs printed for 93% of pts, 89% adherence in placing bed poster above pt’s beds. (2) | 3.15 falls per 1000 pt days in the intervention vs 4.18 falls in the control units (p=0.04) |
| Fife et al. (1984)[^3^](#_ENREF_3) CT | 4 units (1 orthopedic, 1 medical unit) in an 410-bed acute care hospital | All physicians and department heads informed of objectives, nurses instructed in all phases (1) | Risk assessment (tool shown)  vs n/a | 18 nursing intervention such as identification arm band, call box, chart cover, and above wall; orange circle placed on incident report when fall occurred; risk/falls criteria card; and new care plan; audit  vs usual care | 1 | 0 | Weekly audits of selected records to check assessment, identification, and documentation for completeness; criteria cards of discharged pts collected. Data n/a (1) | Within 12 weeks, 12 falls in the intervention vs 16 in the control group (n.s.). Falls in intervention group 61% reduced from same period 1 year pre-intervention |
| Hunderfund et al. (2011)[^4^](#_ENREF_4) CT | A neurology unit and 6 medical units in a tertiary hospital | Nurse coordinators reviewed the assessment process with all residents using a handout; education on process for nurses (1) | Physician fall risk assessment on admission (part of neurology electronic order set) in addition to nurse assessment (Hendrich II Fall Risk), reconciliation, documentation card, education material  vs nurse Hendrich II only, education material | Fall prevention measures as before (fall prevention measures selected by nurse, e.g., assisting with ambulation, call light within reach, pt education, low bed position, side rails, surveillance, bed and chair alarms, sign on door)  vs existing care processes (fall prevention measures selected by nurse, e.g., assisting with ambulation, call light within reach, pt education, low bed position, side rails, surveillance, bed and chair alarms, sign on door) | 2 | 2 | Complete fall risk assessments were performed in 73% of eligible patients. (1) | The fall rates declined significantly in the intervention unit from 5.69 falls per 1000 pt days before to 4.12 whereas the rate of falls on other units did not significantly change. |
| Kilpack et al. (1991)[^5^](#_ENREF_5) Controlled (comparison to total hospital data) B-A | 2 adult medical-surgical units (neuro-science, oncology / renal) in a tertiary care facility | Educational program to increase staff’s cognizance of fall prevention (1) | Fall recorded on Kardex; staff risk awareness posters  vs usual care | Clinical nursing specialist develops written care plan for pts who fell, disseminated at change-of-shift report; risk alert signs in medical record and above bed  vs usual care | 1 | 0 | Clinical nursing specialist checked within 2 days whether recommended interventions had been implemented; number of falls posted in unit; quarterly summary of fall statistics; continuing education. Of the planned interventions, 83% were implemented by the nursing staff. (2) | The fall rates declined from 4.7 per 1,000 pt days to 4.4. The all-hospital fall rate increased from 3.0 per 1,000 pt days to 3.6 in the same time period |
| Krauss et al. (2008)[^6^](#_ENREF_6) CT | 4 general medicine floors in an urban, 1300-bed tertiary-care academic hospital | In-services and self study modules (1) | Daily risk assessment (Morse Fall Scale), pt education, modify environment to make it safer  vs daily risk assessment | Alert signs above bed and/or wrist band; pt / family education; toileting schedule / 2 hour safety rounds; medication review; walking aids; other measures (e.g., bed alarm system, low bed, request that family sit with pt, room near nurses’ station)  vs usual care (many falls prevention strategies already in place, e.g., pt / family education, signs, other measures) | 2 | 2 | Monthly feedback on fall rates, weekly audits of prevention strategies. Significant differences between intervention and control group for bed brakes on, use of wristbands, dot on chart, dot on census board, activity level displayed on board, pt education, toileting schedule maintained, medication discussed, exit alarms. (2) | The fall rates were not statistically significantly different between the intervention and control group (p=0.41); the falls rate in the intervention group dropped from 6.64 falls per 1000 pt days before to 3.81 5 months after the intervention (p=0.04), the change was not sustained after 9 months (p=0.31) |
| Meade et al. (2006)[^7^](#_ENREF_7) CT | 27 nursing units in 14 hospitals | Training session for nursing staff (1) | Two-hour rounding (offering toileting assistance, call light within reach etc.)  vs one-hour rounding  vs usual care | n/a | 1 | 0 | Daily rounding logs and patient reports reviewed by nurse manager.  Data n/a (1) | 13 falls in the 2-hour rounding group after intervention vs 19 before (n.s.); 12 falls in the 1-hour rounding group after vs 25 falls before (p=0.01); control group 19 (pre) and 13 (post) falls |
| Padula et al. (2011)[^8^](#_ENREF_8) CT | 3 medical-surgical units in an acute care teaching hospital (1 intervention, 2 control) | Research nurse trained to implement the protocol (1) | Targeted lower extremity strengthening exercises and ambulation; Geriatric Friendly Environment through Nursing Evaluation and Specific Interventions for Successful Healing mobility protocol  vs ambulation; Geriatric Friendly Environment through Nursing Evaluation and Specific Interventions for Successful Healing mobility protocol without strengthening exercises | n/a | 1 | 1 | Research nurse provided intervention support and monitored documentation. Random record reviews and chart audits validated that the mobility protocol was implemented consistently in intervention and control group. (1) | In the 6-month study period, the mean fall rate in the intervention group was 3.2 per 1000 patient days and 2.8 and 3.3 in the control units |
| Shorr et al. (2010)[^9^](#_ENREF_9) Cluster RCT | 16 general medical-surgical nursing units in a community hospital | n/a (0) | n/a | Proximity alarm systems plus existing fall prevention protocols  vs usual care (existing fall prevention protocols) | 0 | 1 | Daily rounds by team dedicated to promoting and facilitating alarm use. Prevalence of alarm use was 64.41 days / 1000 pt days in treatment, 1.79 days / 1000 pt days in control units. (2) | The intervention group was not significantly different from the control group regarding the rates of falls (p=0.19) or injurious falls (p=0.96) |
| Spetz et al. (2007)[^10^](#_ENREF_10) CT | A post-neurosurgery unit in an acute care hospital | n/a (0) | n/a  vs risk assessment | An electronic pt vigilance system (sensor array measuring pulse and respiration placed under bed, bedside unit connected to nurse call system, bed exist alert)  vs usual care (including pt sitters, assisting with ambulation, moving pts closer to nurses’ station, bedrails, restraints, other standard practices to prevent falls) | 0 | 2 | n/a (0) | 2 falls (1.94%) in the intervention vs 15 falls (3.23%) in the control beds |
| Tideiksaar et al. (1993)[^11^](#_ENREF_11) RCT | A geriatric evaluation and treatment unit in an acute hospital | n/a (0) | n/a | Bed alarm system, hourly checks, restraints  vs usual care (hourly checks, restraints) | 1 | 1 | System was well accepted by pts, family, and staff. The alarm was utilized 4,425 hours, nurses responded to 92% of alarms in less than 1 minute (2) | 1 fall from bed, 5 falls in total in the intervention vs 4 falls from bed and 12 falls in total in the control group (bed falls p=1.00) |
| **Historic controls** |  |  |  |  |  |  |  |  |
| Amato et al. (2006)[^12^](#_ENREF_12) Time series | A stroke rehabilitation unit and brain injury rehabilitation unit in a teaching hospital | Initial and update meetings with key clinical and administrative stakeholders to gain support; education sessions for nursing staff | Post-fall review  vs n/a | Physical restraint reduction program, bed exit alarms, surveillance techniques (15 min checks, moving pts) and assessment rounds, weekly consultation rounds and feedback,  vs before (use of physical restraints) | 1 | 1 | Nurse specialist monitored adherence during weekly rounds and review of monthly run charts for fall rates and physical restraint use. Data n/a (1) | Falls rates decreased from 11.4 to 6.1 (average stroke unit, 45.5% relative reduction) and from 9.1 to 3.3 (average brain injury unit, 64.2% reduction) |
| Barker et al. (1993)[^13^](#_ENREF_13) B-A | 2 psychiatric units in an acute hospital | Falls awareness program, nursing task force established, committee reporting to senior management group, staff education (2) | Risk assessment (tool shown), post-fall review  vs n/a | Standardized nursing care plan for at risk pts, sticker for chart, pt education, Geri chairs, Posey vests, nursing staff mix modified to meet custodial needs, pts needing supervision clustered, toileting and ambulation schedules, status reports in change of shift report, close observation schedules  vs before | 2 | 0 | Falls assessment form added to admission form.  Data n/a (0) | Falls rates decreased from 6.84 to 4.16 per 1,000 pt days after 4 year intervention |
| Beasley et al. (2009)[^14^](#_ENREF_14) Time series | A full-service tertiary community hospital | Policy and procedure presented to pharmacy and therapeutics committee for approval; pharmacist education (1) | Risk assessment with medication review (factors reported)  vs before (risk assessment without medication review) | Daily medication review by pharmacists, medication fall risk score; recommendations communicated to physicians, evaluation placed in pt chart; care plan specifies use of call light for assistance; pt education  vs before (no medication review) | 1 | 1 | Fall prevention handbook part of admission packet; falls data reported to falls and restraints committee twice a year.  Data n/a (0) | The total falls rate decreased from a mean of 4.69 to 1.07 (total fall rate decrease by 30%), the injury fall rate decreased from 2.06 to 1.06 per 1,000 pt days after 2-year implementation (48% reduction) |
| Browne et al. (2004)[^15^](#_ENREF_15) B-A | 5 hospitals in a health care system | Education (fairs for nurses) (1) | Computerized information system (ADAPT Fall Tool)  vs modified Hendrich Fall Assessment tool | IT system tailors interventions to specific risks (disorientation / activity / post-medication / toileting precautions), fall risk problems prioritized on care plan, highlighted on multidisciplinary report sheet, part of patient care conferences  vs before (daily high risk pts report, armbands, signs above bed, sticker in chart; care plan adjustments according to risk, 40 potential interventions to chose from) | 2 | 1 | Assessments integrated into admission and shift assessment documentation. 100% compliance. (2) | Fall rates decreased from 3.41 per 1, 000 adjusted pt days before the intervention to 3.21 after (n.s.). Fall-related injuries per 100 falls decreased from 1.44 to 0.95 (n.s.) |
| Callahan et al. (2009)[^16^](#_ENREF_16) B-A | A hematology-oncology / bone marrow transplant unit | n/a (0) | Hourly nursing rounds (checking for pain, positioning / comfort, toileting, personal needs, safety), medication review, daily risk assessment (Morse Fall Scale)  vs before | n/a | 1 | 0 | Daily weekday huddles with team to evaluate process and address barriers. There was increased staff satisfaction. (1) | Fall rates with injuries decreased by 100% from 3 during 3 months prior to 0 after the intervention |
| Capan et al. (2007)[^17^](#_ENREF_17) B-A | An acute care hospital | Multi-disciplinary task force, root cause analysis, staff involvement in equipment selection, staff education (including self-directed leaning module with test, awareness promoted), unit champion, gift card competition for fall prevention (2) | Risk assessment every 12 h (tool shown, includes Timed Up and Go test); interdisciplinary team rounds to discuss care plans; post-fall review  vs before | Wrist band; sign on door; pt / family education; hip protector; orthostatic hypotension assessment every 24 h; plus interventions based on risk scores (e.g., bed / chair alarm, delirium assessment, prompted toileting); fall risk and interventions part of change of shift report and interdisciplinary plan of care form  vs before | 2 | 0 | Checklist of interventions on same sheet as assessment tool, triggered by score, rounds and shift reports review assessment and interventions. 95% of staff completed the education sessions. (1) | Fall rate dropped from 0.45 per 100 pt days before intervention to 0.32 per 100 pt days after. Severity of fall-related injuries also declined |
| Carroll et al. (2009)[^18^](#_ENREF_18) B-A | An acute care hospital, data for head trauma unit | Fall quality improvement initiative assembled interdisciplinary team; staff education (50 classes for frontline staff) (2) | Risk assessment (tool shown)  vs before | 4 core elements (identification, monitoring, modifying physical environment, pt specific interventions); sign posted on door and assignment board; armband; computer order placed to alert other departments; orders are reflected on printable care plan used for shift reports; clutter eliminated; nursing protocols developed (to choose individual care plans), catalog of interventions (e.g., hourly rounds)  vs before | 2 | 0 | Nurse documentation forms cue interventions. Data n/a (0) | Number of falls and falls with injuries increased initially, then reduced significantly 3 months after intervention implementation. A head trauma unit reported a decrease in fall average of 14.18 to 2 falls per 1000 pt days |
| Cohen et al. (1991)[^19^](#_ENREF_19) B-A | A neuroscience unit in an acute hospital | Discussion with staff, educational campaign (including presentations at staff meetings, knowledge test), part of nursing orientation (1) | Risk assessment; daily classification of pt needs (Nursing Productivity and Quality system)  vs before | Sign placed on door, over bed and Kardex flagged; individual care plan depending on mental status documented on form (e.g., pt and family education, hourly rounds, restraints)  vs before | 1 | 0 | Program compliance and falls rate monitored, feedback; ongoing awareness efforts and project updates.  Data n/a (1) | Fall rates remained ≤ 3.8 per month in 8 out of 12 months within 1 year of implementation; the fall rate was 3.8 before the intervention |
| Croft et al. (1992)[^20^](#_ENREF_20) B-A | A 220-bed hospital | n/a (0) | Risk assessment (factors reported)  vs before | Stickers on beds, doors, nursing care plans, next to names on patient call system; pt / family education; family alerts nurses when leaving; safety room check (bell within reach, side rails, bathroom light on, bed wheels locked, non-slip socks / slippers, restraints); fall evaluation card when falls occur  vs before | 1 | 0 | n/a (0) | Falls decreased by 60% after intervention. |
| Dacenko-Grawe et al. (2008)[^21^](#_ENREF_21) B-A | Acute care 325 bed teaching hospital | Education for nursing staff and ancillary departments (1) | Risk assessment (tool shown) every shift; non-skid footwear; fall review tool after fall occurred (tool shown); hourly rounding established in last year  vs before | Identification bracelets, symbol placed on door; all staff instructed to monitor and accompany pts to the bathroom or bed; bed exit alarm; pt / family education including leaflets; reminder sign (native language) not to get out of bed in pt room; frequent rounds; sitters  vs before | 2 | 0 | Unit manager and charge nurse know score during rounds and can assure implementation of interventions; barriers to implementing protocol assessed. (1) | The falls per 1000 pt days decreased from 4.04 (255 falls) before the implementation to 2.27 (123 falls) after 4 years (p=0.002); the intervention continued to evolve in this period |
| Diduszyn et al. (2008)[^22^](#_ENREF_22) B-A | 3 telemetry floors and 1 neurology floor in a 500 bed acute care teaching hospital | Education for all shifts (1) | Fall report form  vs before | 6 wireless nurse call fall monitors (bed alarm, alarm announces message, e.g., ‘please stay in bed’ and alerts nurse’s beeper); fall report form  vs before (existing falls prevention program including bed alarms, sound in pt room only) | 0 | 1 | At least 1 nurse scheduled who knew the device on each floor for every shift. 91% of survey respondents thought the device helped to prevent falls, 13% of fallers and 16% of high risk pts used the device; many nurses indicated that they were unaware of the availability of the device.(1) | The number of falls decreased by 18% from 78 within 4 months before to 65 falls within 4 months in the same period after the intervention |
| Geffre (2006)[^23^](#_ENREF_23) B-A | 6 medical units (medical, oncology, surgical, telemetry, transitional care, rehabilitation) | Staff education on risk assessment tool and alarm (1) | Risk assessment (factors reported)  vs before | Bed exit alarm  vs before | 0 | 0 | n/a (0) | Incidence rate of falls decreased from 2.04% to 1.52% after the intervention (n.s., 25% decrease), rate of unassisted falls (no caregiver present) decreased by 38%, rate of assisted falls (a caregiver was present and unsuccessfully tried to prevent the fall) went up from 0.11% to 0.32% |
| Gilbert et al (1999)[^24^](#_ENREF_24) B-A | Neuroscience unit, tertiary care university hospital | Educational program on restraints and alternatives (1) | n/a | Restraint reduction program (use of rocking chair, recliner, Ambualarm, mittens); daily rounds to assist staff in developing care plans  vs before (use of physical restraints) | 0 | 1 | n/a (0) | The fall rate was 7.84% during the three month implementation phase and 8.56% for the three months earlier |
| Goodlett et al. (2009)[^25^](#_ENREF_25) B-A | A 34-bed internal medicine unit | Staff education (1) | n/a | 24-hour camera surveillance, monitor observed 24 hours by unlicensed assistive personnel, intervening to prevent falls (going to the bedside, speaking through the call system, alerting other staff, notifying nurses of increased restlessness, agitation or other behavior)  vs before | 0 | 0 | n/a (0) | Mean annual unit fall rate decreased by 6% after implementation, rate in surveillance rooms not lower than in the rest of the unit (p=0.548); only 1 fall in 417 pts occurred in the surveillance rooms in 12 months, 0.68 falls per 1,000 pt days |
| Gowdy et al. (2003)[^26^](#_ENREF_26) B-A | A community hospital | Multidisciplinary fall team established; staff education (in person, posters, educational fair; grant obtained to fund education); innovations piloted in selected areas then spread (2) | Risk assessment (tool shown) every 12 hours’ pt / family education; posters; root cause analysis on each fall, fall cause analysis tool (tool shown)  vs before | Interventions stratified by risk listed on tool (e.g., bracelet, colored name card on door, non-slip socks or shoes, remove clutter, consult pharmacist for medication review); reassessment every 12 hours; bed exit alarms and motion detectors; assistive walking devices; mirrors enable nurses to supervise the hallways  vs before | 2 | 0 | n/a (0) | The fall rate decreased from 6.1 before to 2.6 per 1,000 pt days after the intervention |
| Guarascio-Howard (2011)[^27^](#_ENREF_27) B-A | A medical-surgical unit with 24 rooms in a hospital | Education / training; bed alarm protocol and bed protection template developed with input from management (1) | Wireless communication device equipped with display and audio for team communications and alarms, bed exit alarms and bed status changes displayed as text messages with audible notifications; bed alarm protocol; bed protection template (bed brake on, low bed, head rail up), staffing changes  vs before (fixed communication audio stations, alarms rang at master station and pt room, pillow speaker to communicate with pt, caregiver had to see call light or be notified) | Bed protection template stratified by risk (e.g., bed exit alarm, all 4 bedrails up, physician’s order)  vs before | 0 | 0 | Support system for system failures established; 4 nurses shadowed to collect detailed adherence data. Wireless communicator use was 12%, phone use decreased from 20% to 11%. (2) | Average 6-months fall rate decreased from 2.7 to 2 (n.s.) in the intervention period |
| Gutierrez et al. (2008)[^28^](#_ENREF_28) B-A | A definitive observation unit in a telemetry setting | Multidisciplinary team; PDCA model; timeline disseminated; staff education; team champions recruited to approach and educate staff (‘elevator speech’); train-the-trainer session; staff involved in selecting interventions (2) | Risk assessment (Morse), risk score included in nurse report; post-fall order sets  vs before | Fall protocol; high-fall risk order sets; designated area for high risk pts; low beds; bed alarms; portable computers for documentation within the sight of pts  vs before | 1 | 0 | Compliance with interventions audited, team rounded to ensure compliance, falls monitored, barriers assessed. >50% of surveyed RNs had experienced a pt fall in the last year but were not communicating the fall-risk level at report or during transfer. (1) | Fall rate decreased from 4.87 per 1000 pt days to 3.59 immediately after the intervention and to 1.37 3 months later |
| Hendrich (1988)[^29^](#_ENREF_29) B-A | A hospital | Hospital committee addresses falls, staff encouraged to participate and input / involvement sought, pilot units before full rollout; staff training, flyer sent to all hospital employees (2) | Risk assessment (Hendrich, tool shown); post-fall form (tool shown)  vs before | Wrist bands, symbol above bed and charts, pts placed close to nursing station, assistance with bathroom rounds, bedside commodes, night lights in room and bathroom, low bed, footwear, supervision while out of bed; reassessment every 8 hours; bed exit alarm, pt / family education  vs before (e.g., night lights, rounds, side rails) | 2 | 1 | Assessment added to admission form; flow sheet to document interventions. Data n/a (0) | Falls decreased by 50% compared to the same months in the pre-intervention year |
| Hernandez & Miller (1986)[^30^](#_ENREF_30) B-A | A gero-psychiatric unit in a medical center | Staff education, assumptions about fall causes tested and shared to keep staff informed, interested and supportive of the project (1) | Risk assessment (tool shown)  vs. before | Three levels of fall precautions and related interventions (e.g. call lights pinned to pt gowns signals bed exit, pt education, regular safety rounds, group high-risk pts together during periods of staff shortage)  vs. before | 1 | 0 | n/a (0) | Before the intervention, pts fell at a rate of 24.98 per 1000 pt days; the fall rate decreased by 42.3% in the 1^st^ intervention year and another 39.4% in the second year; a total decrease of 81.75% |
| Innes & Turman (1983)[^31^](#_ENREF_31) B-A | An acute care, 362 bed hospital | Multidisciplinary workshop to select interventions, pilot unit before rollout; staff training (2) | Risk assessment (St Francis, tool shown)  vs before | Stickers for door, call button in nurse station and Kardex; poster to encourage pts to call for help; pt / family education  vs before (physical restraints, medication) | 1 | 1 | Risk assessment added to patient record, includes intervention recommendations; monitor sheets to track preventive measures. Nursing staff survey showed that the identification, the highlighted Kardex and call button were considered useful. (1) | 3 falls occurred in a 725 pt day period after the intervention implementation, 6 falls in 730 pt days were recorded previously |
| Innes (1985)[^32^](#_ENREF_32) B-A | An acute care hospital | Multi-disciplinary workshop to re-educate staff and discuss barrier to implementing existing program (1) | Risk assessment (St Francis, tool shown)  vs before | Stickers for doors, nursing call buttons and Kardex; poster to encourage pts to call for help; pt / family education; ambualarm  vs before (stickers for doors, nursing call buttons and Kardex; poster to encourage pts to call for help; pt / family education) | 1 | 1 | Bi-monthly performance feedback; annual workshop; periodic re-education (including video tape) for all staff. Data n/a. (1) | The number of falls was reduced 44% after 1 year and the ratio of falls to pt days decreased 37.5% |
| Karius et al. (2006)[^33^](#_ENREF_33) B-A | Inpt oncology unit | A multi-disciplinary team was assembled (1) | Risk assessment (Hendrich II Fall Risk); pt and family education including posters in pt rooms and leaflets, daily pharmacy reviews of medication profiles for high-risk medications; physical therapy department reviews fall risk documentation  vs before (different risk assessment tool) | Collaborative prevention strategies  vs before | 1 | 1 | Education leaflet added to admission packet.  Data n/a (0) | The number of falls decreased from 48 before to 37 after the intervention and remained stable the following year. No severe fall related injury happened after the intervention |
| Kolin et al. (2010)[^34^](#_ENREF_34) Time series | Rural medical center within health care system with acute care facilities | Redesign team established; rapid improvement event (interdisciplinary workshop, groups formed); work groups develop and test new strategies, analyze data and plan changes; education for all nursing staff and staff fact sheets (2) | Risk assessment (tool shown); basic safety interventions (bed locked, in low position, call bell in reach); pt and family education brochure and DVD; post-fall form with mini-root cause analysis (tool shown)  vs before (different risk assessment tools) | Medium risk: basic safety interventions plus arm band; rounds with checklist; bed alarms based on clinical judgment; high risk: plus mandatory bed alarms and appropriate room location  vs before | 2 | 1 | Compliance with recommended intervention implementation and post-fall documentation tested using medical record audits.(1) | In one medical center, falls decreased from 21 to 5 per month after 6 months of intervention; previously 174 falls and 3 falls with injury. Injury rate of 1.7 reduced to an average fall rate per 1000 pt days of 2% and 0 injury rate per 10,0000 pt days in the 3 months after the intervention |
| Kratz (2008)[^35^](#_ENREF_35) B-A | Medical-surgical unit in a medical center | Education for nursing staff (self-learning packet, monthly discussions and education at staff meeting, information in new hire orientation) and presentations at physician departmental meetings (2) | Acute confusion protocol with interventions aiming at orientation (e.g. use reminiscence), non-pharmacologic sleep (e.g., use soft music), and early mobilization (e.g., use toileting schedules); pharmacist sends alerts for medications on Beers list, standard order set revised, RNs suggest alternatives when noticing listed medications  vs before | n/a | 1 | 0 | Project information in new hire orientation. Data n/a (0) | Pt fall rate decreased from 4.8 per 1000 pt days to 3.6; decrease has been maintained for 3 years |
| Lancaster et al. (2007)[^36^](#_ENREF_36) B-A | 9 hospitals in a health system | Alpha sites with interdisciplinary teams tested strategies; hospitals joined voluntary falls affinity group with monthly conferences to learn from each other (1) | Risk assessment (Hendrich II Fall Risk)  vs before | Visual identification; communication of risk status; pt, families and staff education; specific interventions (e.g. sign on door, non-skid slipper socks, sticker on chart) varied by site  vs before | 1 | 0 | Appr. 80% of hospitals joined the falls affinity group. (0) | Acute fall rates were reduced 9.9% after intervention |
| Lane et al. (1999)[^37^](#_ENREF_37) B-A | Medical-surgical / critical care units in a metropolitan community hospital | Program originally developed through pilot study (7 years earlier) (0) | Risk assessment (factors reported)  vs before | New care guideline  vs before | 1 | 0 | Fall risk assessment completed during admission process for all pts. (2) | Fall rates increased from 2.27 per 1000 pt days (412 falls / 181,876 patient days) before intervention to 3.89 after (373 falls / 95,867 patient days) |
| Llewellyn et al. (1988)[^38^](#_ENREF_38) B-A | An acute cardio-vascular surgery unit in a medical center | Safety committee formed; comprehensive, in-service education session (1) | Risk assessment (tool shown)  vs before | Interventions selected based on assessment (sticker, environment check at end of shift, blood pressure check at admission, scheduled toileting, side rails, rounding, no sleeping medications, call light pinned to gown, restraints), reinforce interventions during shift report; fall review  vs before | 2 | 0 | Interventions documented on assessment form.  Data n/a (0) | The rate of falls increased from 3.4 to 4.4 per month in the 1^st^ year and 3.8 per month in the 2^nd^ year of implementation |
| McCollam et al. (1995)[^39^](#_ENREF_39) B-A | 40-bed cardiology general medicine unit in a VA hospital | Education (including fall risk assessment video), approval by nursing administration; intervention piloted in selected unit (2) | Daily risk assessment (Morse Fall Scale)  vs before | Interventions based on assessment  vs before | 0 | 0 | Fall risk assessment added to admission assessment. Follow-up monitoring of compliance with instrument completion on admission ranged from 75% - 85% in the 18 months after introducing; high risk interventions only present in 50 – 58%. (2) | Before the intervention, there were 186 falls and 11 related serious injuries. After, there were 245 falls and 4 serious injuries |
| Meissner et al. (1988)[^40^](#_ENREF_40) B-A | A medical, 35 bed unit in a hospital | Staff education and awareness training (including mandatory in-service sessions, video annually reviewed) (1) | Risk assessment  vs before | Written care protocol; interventions depend on assessment (e.g. bed alarm; hourly safety assessments; signs on door and intercom panel; restraints; sitters)  vs before | 2 | 0 | Educational video annually reviewed (0) | The rate of falls with related injuries decreased by 100% after the intervention (from 10 to 0 falls 6 months post-intervention) |
| Miller et al. (2008)[^41^](#_ENREF_41) B-A | A leukemia-lymphoma unit in a hospital | Unit committee; staff re-education (1) | Hourly rounding (including toileting, pain assessment)  vs before | Existing interventions (sign on door (sign shown), identification bracelet, pt education, checklist with nursing interventions (e.g. use of bed exit alarm), high-risk medications and alternatives listed); audit and feedback of rounds  vs before (existing fall prevention packet) | 2 | 2 | Rounds were documented and checked during first month.  Data n/a (0) | The intervention reduced the average fall rate from 4.88 per 1,000 pt days to 3.28. The injurious fall rate decreased from 1.2 to 0.94 |
| Mion et al (2001)[^42^](#_ENREF_42) B-A | 2 medical, 1 neurological and 1 surgical unit of an urban academic medical center (1,000 beds acute care hospital) | CQI approach; interdisciplinary team gained support from clinical and administrative personnel; group education (mandatory for nursing personnel) (2) | Restraint reduction program (administrative, educational, consultative, and feedback component); interdisciplinary rounds; delirium assessment, prevention and management strategies; anchoring / securing therapeutic devices  vs. before | Falls prevention strategies (e.g. increased surveillance by moving pt to room within view of nurses’ desk, toileting schedule); case conferences  vs before | 1 | 0 | Daily consultative rounds with audit and feedback on strategies; fall rates presented monthly.  Data n/a (1) | In the same time period units had falls rates of 0.2, 0.3, 0.4, and 0.3 per 100 pt days before the intervention, all units were reduced to 0.1 after the implementation |
| Morton et al. (1989)[^43^](#_ENREF_43) B-A | A 42-bed unit in a hospital | Program integral part of staff orientation (1) | Risk assessment (factors reported)  vs before | High-risk note on admission sheet, Kardex, nursing notes, intercom console; sticker on pt chart; sign near door; frequent checkups; bed rails; call light within reach; low bed position; supervised while out of bed or special equipment; non-skid footwear; bedside commode; nightlight; pt and family education; fall risk part of shift report; bed alarm (Bedcheck)  vs before | 2 | 0 | Integral part of staff orientation and evaluation; compliance monitored, program is deliberately kept simple; fall-free days posted, celebration for shift that achieves fewest falls per quarter.  Data n/a (1) | The fall rate dropped 25% in the 1^st^, 8% in the 2^nd^ year; after introducing the bed alarm, falls were reduced 47% after 1^st^ year, 60% after 2^nd^ year. Recurrent falls dropped 29% after 2 years |
| Mosley et al. (1998)[^44^](#_ENREF_44) B-A | A VA hospital | Structured education in-service (1 h / week for 4 weeks during every shift) for nursing staff; information memo to all service chiefs and request for cooperation ; phased in at 3-month intervals for different units (2) | Initial assessment during admission  vs before | Risk assessment (Fall Assessment Form, Berryman), reassessment after changes / every month; risk and interventions noted on plan of care; several interventions (e.g. sticker on chart, bed, nursing care plan, identification bracelet; pt and family education; low bed; bed rails, close supervision, nonskid footwear, clear paths, encourage buddy system, medication assessment  vs before | 2 | 0 | All 16 fallers were correctly identified as at risk and prevention protocol had been implemented. (1) | Mean fall rates decreased from 7.07 before to 6.33 after (p<0.003); 6 months after the study an additional 35% decrease in the number of falls was observed |
| Neiman et al. (2011)[^45^](#_ENREF_45) Time series | A pediatric hospital | Program tested in pilot units; staff education; newsletter described program (1) | Electronic risk assessment (I’M SAFE Fall Risk Assessment Tool, tool shown) every shift; high-risk interventions for all intensive care units and children < 2 years; family education; low bed; side rails; bed brakes on, clutter minimized  vs. before | Interventions based on risk documented in electronic record (medium: assisting with activities, periodic assessment of elimination needs; periodic call light orientation; high: observation, assistance when out of bed; sign at bedside)  vs before | 2 | 0 | Tool integrated into electronic medical record nursing documentation workflow; program incorporated in nursing orientation.  Data n/a (1) | Intrinsic fall rates declined from 0.67 before the intervention to 0.51 per 1,000 pt days after (p=0.015) |
| Peterson et al. (2005)[^46^](#_ENREF_46) 2 inter-vention (after) and 2 control periods (before) | Medical, surgical, neurology, and gynecology services of an urban 720-bed tertiary care hospital | n/a (0) | Medication decision support tool (adapts dose for elderly pts, suggests alternative psychotropic medication)  vs before (usual computerized order entry) | n/a | 0 | 0 | Tool part of electronic order entry (dose defaults changed). Prescriptions agreed more often with recommendations (29.3 vs 19.4%, p<0.001). (2) | Patients in the intervention cohort had a lower in-hospital fall rate (0.28 vs 0.64 falls per 100 pt days; p=0.001) |
| Quigley et al. (2009)[^47^](#_ENREF_47) Time series | 2 units (general medicine and oncology; medical-surgical and respiratory care), 34-bed and 21-beds, in VA hospital | Interventions tested by pilot teams, small tests of change; engaging unit-based staff in change; nurse managers trained staff in safety huddle process through role-playing, brochures and a presentation (2) | Risk assessment (factors reported); post-fall safety huddles (staff, pt and family); comfort care and safety rounds; pt education  vs before | Pt education (teach back method); toileting prior to pain medication; pts with risk factors placed on high-risk precautions (e.g., moving pt close to nurse station, chair / bed alarm, hourly rounds, nonskid socks, wristbands and other visual identifiers); injury risk prevention (e.g., hip protectors)  vs before | 2 | 0 | Falls precautions incorporated into handoff process (electronic report plus 1-minute joint assessment at bedside). 66% of pts able to teach back fall risk factors, all pts able to teach back benefits of asking for help and to demonstrate how to call nurse; pts continued to get up to go to the bathroom despite the toileting intervention. (2) | The fall rates slightly decreased after the intervention (3.62. to 2.78). The fall related injuries remained stable |
| Rainville et al. (1984)[^48^](#_ENREF_48) B-A | Medical surgical units in a 248-bed facility | Nursing committee designed standard care plans for high-risk pts; nurses instructed in use of care plan (1) | Risk assessment (St Francis; factors reported) within 24 hours of admission, repeated weekly  vs before (inconsistent risk assessment) | St Francis care plan implemented, care plan lists interventions (tool shown); pt and family education; environment (bed rails, low bed, call light within reach, room light, nonskid footwear, assisting in voiding every 4 hours); staff awareness (care plan kept with Kardex, sign over bed)  vs before (inconsistent risk assessment, restraints, medication, relocation closer to nurse station, family sitter) | 1 | 1 | n/a (0) | 26 falls (average daily census 109.3 pts) before versus 27 falls (average daily census 118.5) after intervention implementation; in pilot unit, falls decreased by 10% after the intervention with a 4% increase in pts days (from 3351 to 3488) |
| Rauch et al. (2009)[^49^](#_ENREF_49) B-A | A unit in an academic medical center | Multidisciplinary team revised policy, involvement of outside consultant; mandatory staff education according (including needs assessment) for all shifts; pilot units tested and revised interventions before rollout; fall prevention unit champion determined; PDSA model applied; active leadership role, staff fully engaged (2) | Risk assessment (Schmid Fall Risk Assessment Tool); pt education  vs before (existing policy, no risk assessment tool) | Interventions listed on back of risk assessment tool (pocket card), stratified by risk; visual identifiers (stickers on doors, white board in nurse station, pt bracelet), documentation (electronic chart), pharmacy evaluation of medications; list of at risk pts printed daily; rounding sheet for documentation of at risk pts; post-fall protocol  vs before (existing policy) | 2 | 1 | Assessment tool chosen that allows rapid completion, computerized (nurse receives list of high risk pts, previous data carried forward automatically); pt education part of admission packet; meetings to ensure compliance; bi-monthly gap analysis (tool used to measure compliance and success). 100% correct use of identifiers, rounding sheet, alarms, placement near station in pilot unit; program was well received by staff. (2) | The rate of falls with injury decreased from 43 to 14% after the intervention |
| Ruckstuhl et al. (1991)[^50^](#_ENREF_50) B-A | A 1,145 bed acute care medical center | n/a (0) | Risk assessment (tool shown); interventions include low bed, call light within reach, calls need to be answered promptly, assistive devices within reach, skid proof footwear; eliminate hazards, educate about roll table, pt orientation to surrounding  vs before (existing protocol, tool with 5 levels of risk, reassessment every shift) | Interventions stratified by risk level (level II: side rails, instruct pt and family to ask for assistance; all items within reach; level III: night light; reorientation; observation; assistance; restraints; sitter)  vs before (existing protocol, interventions also stratified by risk) | 2 | 1 | Interventions listed on risk assessment tool; tool streamlined to allow quick assessments.  Data n/a (0) | Falls resulting in fractures decreased by 33% after 1 year and 89% after 2 years of implementation |
| Schmid et al. (1990)[^51^](#_ENREF_51) B-A | A 700 bed government-owned medical center | Program reminders through daily news bulletin; education for devices for nurses on all shifts (1) | Risk assessment (Schmid Fall Risk Assessment Tool, tool shown) at admission, then weekly or after status changes  vs before | New nursing care plan, interventions based on risk; signs over bed and outside room; staff is asked to notify nurses in unsafe situations; form to document precautions; more bed alarms; safety vests; falls documented in incidence reports; falls per month monitored  vs before (e.g., older safety vests, fewer alarms) | 2 | 1 | Self-monitoring of accuracy of risk scores and compliance at nursing unit level; compliance assessed for high risk pts; later risk assessment included in Kardex. Compliance with program ranged from 50 to 100% per month before new forms were introduced. (2) | Within 3 months after the program, the fall per pt day ratio dropped to 27 from 50 falls per 10,000 pt days (54% decline), data fluctuate; 1 year after the implementation the reduced incidence of pt falls is maintained (41 falls / 10,000 pt days in 4 months); in the 12 months following the intervention implementation monthly fall rates per pt day have averaged 20% lower than peak levels previously |
| Stuart et al. (2010)[^52^](#_ENREF_52) B-A | A neuro / stroke unit in an academic medical center | Performance improvement team; PDSA model (2) | Rescheduled routines so staff is available at high risk periods; assessment rounds; reference cards for float personnel, warning signs, pt care standards  vs before | n/a | 1 | 0 | n/a (0) | The average monthly fall rate decreased from 2.91 before to 1.67 during the 90 day intervention period |
| Szumlas et al. (2004)[^53^](#_ENREF_53) Time series | A tertiary care academic medical center | Multidisciplinary task force; fall prevention incorporated into organizational strategic plan, program goals communicated by senior leadership, education and awareness training; mandatory 1 hour training session for care managers and services managers, managers trained staff; material distributed and presented at meetings, video; task force available for consultation; risk assessment tool piloted in selected units (2) | Computerized risk assessment tool, every 24 hours (tool shown); orientation to room, call light, falls prevention; low bed, wheels locked; clutter and spill free room; items within reach; adequate lighting; pt and family education (verbal and written material); documentation of education; unit level fall reporting  vs before (existing program including educational material for pts) | Sign on door (with intervention reminder); staff must remain with pt when assisted to bathroom; toileting assistance offered or hourly bedpan; physical and occupational therapy consultation and discharge planning; hourly rounds; sitter if necessary  vs before (existing program including staff education, catalog of 36 interventions) | 2 | 2 | Continuous support from leadership and management; risk assessment incorporated into routine medical record documentation; tools modified to remove barriers; clear unit-specific data and feedback to staff; information incorporated in staff orientation.  Data n/a (1) | 20% reduction in total falls achieved after the intervention (p<0.0001); 15% reduction in average fall rate per 1000 pt days (p<0.0001); average days between falls with injury more than doubled, sustained through first 11 months following implementation |
| Tzeng et al. (2008)[^54^](#_ENREF_54) B-A | 2 acute adult medical units in a hospital | n/a (0) | Patient Attendant Assessment Tool (assessment of pts’ needs for sitters, tool shown)  vs before (no assessment tool) | Sitters, restraints, side rails  vs before (sitters, restraints, side rails) | 0 | 1 | n/a (0) | The mean fall rate per 1,000 pt days decreased from 4.75 (SD 0.74) to 4.35 (SD 0.51) in 1 unit and from 5.13 (SD 1.02) to 4.15 (SD 0.83). Injuries from falls per 1000 pt days increased from a mean of 0.25 (SD 0.25) to 0.59 (SD 0.18) and 0.49 (SD 0.68) to 0.58 (SD 0.83) in the 2 units |
| Ward et al. (2004)[^55^](#_ENREF_55) B-A | A transitional care unit in an acute care facility | FOCUS (Find an improvement opportunity, Organize a team that knows the process, Clarify current knowledge of the process, Uncover root cause of the process variation, Start the improvement cycle)-PDCA approach; interdisciplinary team; staff education; interventions added incrementally (2) | Risk assessment (factors reported); reminder signs for pts to ask for assistance when getting out of bed; pt and family education (including flyer); reports on new admissions include fall history; fall risk included in shift-to-shift report; monthly falls documented in staff room; shift reports during walking rounds; post-fall review with tool and with family conference  vs before | Guardian program (badge indicates who is looking after the pt, sitter); bed alarm; scheduled toileting; safety plan after fall posted near bed  vs before | 2 | 0 | Information added to admission packet. Some falls occurred because new staff was unaware of the program, later incorporated in staff orientation. (0) | The fall index (number of pt falls per month divided by total pt days multiplied by 1000) decreased by 54% from 9.32 before intervention to 4.28 after 1 year (same quarter compared) |
| Wayland et al. (2010)[^56^](#_ENREF_56) B-A | A community hospital | Fall prevention committee; training, new material (1) | Risk assessment (Hendrich II Fall Risk); walking reports at change of shift; posters; documentation of pt and family education; fall risk ranking evaluated biweekly; post-fall review  vs before (Hendrich II Fall Risk tool; interventions chosen according to risk level) | Existing interventions (ranging from sign on door, yield sign instructing pt to call for assistance, to full-time sitter) plus toileting rounds; reminder sign in room to call for assistance  vs before (interventions ranging from sign on door to full-time sitter) | 2 | 2 | Run charts distributed monthly to increase staff awareness; falls monitored; fall prevention training incorporated in nursing orientation.  Data n/a (1) | The fall rates decreased from 4.37 per 1000 pt days before to 1.29 after 1 month and 0 after 2 months of implementation |
| Weinberg et al. (2011)[^57^](#_ENREF_57) Time series | A 714-bed tertiary care teaching hospital | Falls committee; mandatory staff education (videos, hands-on training); enhancing awareness; continuous quality improvement model; involvement of top management; culture change (safety awareness, critical thinking, multilateral collaboration, programmatic ownership, accountability) (2) | Risk assessment (tool shown) but nurses can override scores; post-fall review with documentation of circumstances and future measures including root cause analysis; pt and family orientation  vs before | Medication adjustments after falls, restricted use of sleeping aids; bed and chair alarms; scheduled toileting with documentation  vs before (existing fall prevention program but low compliance) | 2 | 1 | Staff was monitored to ensure proper risk assessment and prevention; regular meetings; pt care unit managers and staff were made accountable for protocol breaches and counseled for repeated noncompliance; daily contests for fall prevention; daily fall prevention rounds; monthly unit-specific fall reports sent to managers.  Data n/a (1) | Fall rates decreased 63.9% (p<0.0001). Minor and moderate fall related injuries decreased by 54.4% and 64.0%, respectively |
| Widder et al. (1985)[^58^](#_ENREF_58) B-A | Orthopedic and general medical units in an acute care hospital | Nurse education (1) | Risk assessment (factors reported)  vs before | Bed alarm (Ambularm) secured above the knee of pt  vs before (e.g., restraints) | 0 | 1 | Nurses were uniformly enthusiastic about the device. (0) | Falls decreased by 45% in the general unit and 33% in the orthopedic unit |
| Zepp et al. (1991)[^59^](#_ENREF_59) B-A | A hospital and medical center | Fall committee; hospital-wide awareness promotion including personal contact with all department directors and information in newsletters; piloted in selected units (2) | Risk assessment  vs before | Sign on door (sign shown), sticker on chart and Kardex; interventions as necessary including placement in room close to nurses’ station, use of sitter; daily reevaluation  vs before | 2 | 0 | Periodic program reminders in newsletters; ongoing monitoring of falls.  Data n/a (1) | 55% decrease in falls in pilot units compared to 16% increase in other units; number of falls dropped 50% after 1-year implementation and a further 39% after the 2^nd^ year |

Notes:

Abbreviations: B-A: Before-After study; CT: nonrandomized controlled trial; RCT: randomized controlled trial; C: comparator; IC: intervention components; n/a: not available, not reported; n.s.: not statistically significant; PI: principal investigator; pts: patients; PDCA: Plan do, check, act model; PDSA: Plan-Do-Study-Act; vs: versus

Implementation score (provided in parentheses following description of implementation): We rated the intensity of the implementation strategy as low (not reported), medium (description of limited implementation strategy e.g., education sessions), or high (e.g., approach developed as part of a continuous quality improvement process).

Intervention component score: The intervention complexity was rated as low (intervention consisted of only one or two selected components), medium (a number of different care processes), or high (several care processes described plus technology support such as bed exit alarms or intense components such as sitters).

Comparator score: We rated the reported comparator information (usual care, before introduction of intervention) as low (no information), medium (some fall prevention measures in place), or high (intensive or complex existing falls prevention approach as comparator).

Adherence score (provided in parentheses following description of adherence stategies and fidelity data): Adherence levels were rated as ranging from low (no strategy reported), to medium (e.g., mechanism to ensure adherence described), to high (adequate fidelity demonstrated by reporting adherence data or describing a mechanism that makes adherence mandatory in clinical practice).

**Online References:**

1. Cozart HCT. Environmental effects on incidence of falls in the hospitalized elderly. Texas Woman's University. 2009;122.

2. Dykes PC, Carroll DL, Hurley A, et al. Fall prevention in acute care hospitals: a randomized trial. JAMA. Nov 3 2010;304(17):1912-1918.

3. Fife DD, Solomon P, Stanton M. A risk/falls program: code orange for success. Nurs Manage. Nov 1984;15(11):50-53.

4. Hunderfund AN, Sweeney CM, Mandrekar JN, et al. Effect of a multidisciplinary fall risk assessment on falls among neurology inpatients. Mayo Clin Proc. Jan 2011;86(1):19-24.

5. Kilpack V, Boehm J, Smith N, Mudge B. Using research-based interventions to decrease patient falls. Appl Nurs Res. May 1991;4(2):50-55.

6. Krauss MJ, Tutlam N, Costantinou E, et al. Intervention to prevent falls on the medical service in a teaching hospital. Infect Control Hosp Epidemiol. Jun 2008;29(6):539-545.

7. Meade CM, Bursell AL, Ketelsen L. Effects of nursing rounds: on patients' call light use, satisfaction, and safety. Am J Nurs. Sep 2006;106(9):58-70; quiz 70-51.

8. Padula CA, Disano C, Ruggiero C, et al. Impact of lower extremity strengthening exercises and mobility on fall rates in hospitalized adults. J Nurs Care Qual. Jul-Sep 2011;26(3):279-285.

9. Shorr RI, Chandler AM, Kessler LA, et al. Trial of Proximity Alarms to Prevent Patient Falls in Hospitals. Journal of the American Geriatrics Society. Apr 2010;58:103-104.

10. Spetz J, Jacobs J, Hatler C. Cost effectiveness of a medical vigilance system to reduce patient falls. Nurs Econ. Nov-Dec 2007;25(6):333-338, 352.

11. Tideiksaar R, Feiner CF, Maby J. Falls prevention: the efficacy of a bed alarm system in an acute-care setting. Mt Sinai J Med. Nov 1993;60(6):522-527.

12. Amato S, Salter JP, Mion LC. Physical restraint reduction in the acute rehabilitation setting: a quality improvement study. Rehabil Nurs. Nov-Dec 2006;31(6):235-241.

13. Barker SM, O'Brien CN, Carey D, Weissman GK. Quality improvement in action: a falls prevention and management program. Mt Sinai J Med. Oct 1993;60(5):387-390.

14. Beasley B, Patatanian E. Development and Implementation of a Pharmacy Fall Prevention Program. Hosp Pharm. 2009;44:1095-1102.

15. Browne JA, Covington BG, Davila Y. Using information technology to assist in redesign of a fall prevention program. J Nurs Care Qual. Jul-Sep 2004;19(3):218-225.

16. Callahan L, McDonald S, Voit D, et al. Medication review and hourly nursing rounds: an evidence-based approach reduces falls on oncology inpatient units. Oncology Nursing Forum. 2009;36(3):72.

17. Capan K, Lynch B. Reports from the field: patient safety A hospital fall assessment and intervention project. Journal of Clinical Outcomes Management. 2007;14(3):155-160.

18. Carroll D, Pappola L, McNicoll L. Fall prevention interventions in acute care settings: the Rhode Island Hospital experience. Med Health R I. Aug 2009;92(8):280-282.

19. Cohen L, Guin P. Implementation of a patient fall prevention program. J Neurosci Nurs. Oct 1991;23(5):315-319.

20. Croft W, Foraker S. Working together to prevent falls. RN. Nov 1992;55(11):17-18, 20.

21. Dacenko-Grawe L, Holm K. Evidence-based practice: a falls prevention program that continues to work. Medsurg Nurs. Aug 2008;17(4):223-227, 235; quiz 228.

22. Diduszyn J, Hofmann MT, Naglak M, Smith DG. Use of wireless nurse alert fall monitor to prevent inpatient falls. Journal of Clinical Outcomes Management. 2008;15(6):293-296.

23. Geffre S. Fall prevention study: bed alarms, investigating their impact on fall prevention and restraint use. *Bed-Check Corporation* 2006; <http://www.bedcheck.com/fall-prevention-study.html>.

24. Gilbert M, Counsell C. Planned change to implement a restraint reduction program. J Nurs Care Qual. Jun 1999;13(5):57-64.

25. Goodlett D, Robinson C, Carson P, Landry L. Patient safety Focusing on video surveillance to reduce falls. Nursing. 2009;39(2):20-21.

26. Gowdy M, Godfrey S. Using tools to assess and prevent inpatient falls. Jt Comm J Qual Saf. Jul 2003;29(7):363-368.

27. Guarascio-Howard L. Examination of wireless technology to improve nurse communication, response time to bed alarms, and patient safety. HERD. Winter 2011;4(2):109-120.

28. Gutierrez F, Smith K. Reducing falls in a Definitive Observation Unit: an evidence-based practice institute consortium project. Crit Care Nurs Q. Apr-Jun 2008;31(2):127-139.

29. Hendrich AL. An effective unit-based fall prevention plan. J Nurs Qual Assur. Nov 1988;3(1):28-36.

30. Hernandez M, Miller J. How to reduce falls. Geriatr Nurs. Mar-Apr 1986;7(2):97-102.

31. Innes E, Turman W. Evaluation of patient falls. QRB. 1983;9(2):30-35.

32. Innes EM. Maintaining fall prevention. QRB Qual Rev Bull. Jul 1985;11(7):217-221.

33. Karius D, Shane C, Rush L, Hubman M. Improving patient safety: implementation of a falls assessment tool and interventions specific to hospitalized oncology patients Oncology Nursing Society 31st Annual Congress podium and poster abstracts. Oncology Nursing Forum. 2006;33(2):472.

34. Kolin MM, Minnier T, Hale KM, et al. Fall initiatives: redesigning best practice. J Nurs Adm. Sep 2010;40(9):384-391.

35. Kratz A. Use of the acute confusion protocol: a research utilization project. J Nurs Care Qual. Oct-Dec 2008;23(4):331-337.

36. Lancaster AD, Ayers A, Belbot B, et al. Preventing falls and eliminating injury at Ascension Health. Jt Comm J Qual Patient Saf. Jul 2007;33(7):367-375.

37. Lane AJ. Evaluation of the fall prevention program in an acute care setting. Orthop Nurs. Nov-Dec 1999;18(6):37-43.

38. Llewellyn J, Martin B, Shekleton M, Firlit S. Analysis of falls in the acute surgical and cardiovascular surgical patient. Appl Nurs Res. Nov 1988;1(3):116-121.

39. McCollam ME. Evaluation and implementation of a research-based falls assessment innovation. Nurs Clin North Am. Sep 1995;30(3):507-514.

40. Meissner BA. Patient fall prevention. Nurs Manage. Jun 1988;19(6):78.

41. Miller L, Limbaugh CM. Applying evidence to develop a medical oncology fall-prevention program. Clin J Oncol Nurs. Feb 2008;12(1):158-160.

42. Mion LC, Fogel J, Sandhu S, et al. Outcomes following physical restraint reduction programs in two acute care hospitals. Jt Comm J Qual Improv. Nov 2001;27(11):605-618.

43. Morton D. Five years of fewer falls. Am J Nurs. Feb 1989;89(2):204-205.

44. Mosley A, Galindo-Ciocon D, Peak N, West MJ. Initiation and evaluation of a research-based fall prevention program. J Nurs Care Qual. Dec 1998;13(2):38-44.

45. Neiman J, Rannie M, Thrasher J, et al. Development, implementation, and evaluation of a comprehensive fall risk program. J Spec Pediatr Nurs. Apr 2011;16(2):130-139.

46. Peterson JF, Kuperman GJ, Shek C, et al. Guided prescription of psychotropic medications for geriatric inpatients. Arch Intern Med. Apr 11 2005;165(7):802-807.

47. Quigley PA, Hahm B, Collazo S, et al. Reducing serious injury from falls in two veterans' hospital medical-surgical units. J Nurs Care Qual. Jan-Mar 2009;24(1):33-41.

48. Rainville NG. Effect of an implemented fall prevention program on the frequency of patient falls. QRB Qual Rev Bull. Sep 1984;10(9):287-291.

49. Rauch K, Balascio J, Gilbert P. Excellence in action: developing and implementing a fall prevention program. J Healthc Qual. Jan-Feb 2009;31(1):36-42.

50. Ruckstuhl MC, Marchionda EE, Salmons J, Larrabee JH. Patient falls: an outcome indicator. J Nurs Care Qual. Oct 1991;6(1):25-29.

51. Schmid NA. 1989 Federal Nursing Service Award Winner. Reducing patient falls: a research-based comprehensive fall prevention program. Mil Med. May 1990;155(5):202-207.

52. Stuart D, Rairigh-Wolfenbarger J, Jennings C, et al. Reducing Falls in the Stroke Population of an Acute Care Hospital: A Performance Improvement Approach. Stroke. Apr 2010;41(4):E255-E255.

53. Szumlas S, Groszek J, Kitt S, et al. Take a second glance: a novel approach to inpatient fall prevention. Jt Comm J Qual Saf. Jun 2004;30(6):295-302.

54. Tzeng HM, Yin CY, Grunawalt J. Effective assessment of use of sitters by nurses in inpatient care settings. J Adv Nurs. Oct 2008;64(2):176-183.

55. Ward A, Candela L, J. M. Developing a unit-specific falls reduction program. J Healthc Qual. 2004;26(2):36-40.

56. Wayland L, Holt L, Sewell S, et al. Reducing the patient fall rate in a rural health system. J Healthc Qual. Mar-Apr 2010;32(2):9-14; quiz 14-15.

57. Weinberg J, Proske D, Szerszen A, et al. An inpatient fall prevention initiative in a tertiary care hospital. Joint Commission Journal on Quality & Patient Safety. 2011;37(7):317-325.

58. Widder B. A new device to decrease falls. Geriatr Nurs. Sep-Oct 1985;6(5):287-288.

59. Zepp S. BAN "A" FALL: a nursing innovation to reducing patient falls. Kans Nurse. Aug 1991;66(7):13.

60. Myers H, Nikoletti S. Fall risk assessment: a prospective investigation of nurses' clinical judgement and risk assessment tools in predicting patient falls. Int J Nurs Pract. Jun 2003;9(3):158-165.

61. Myers H. Hospital fall risk assessment tools: a critique of the literature. Int J Nurs Pract. Aug 2003;9(4):223-235.

62. Harvey K, Kramlich D, Chapman J, et al. Exploring and evaluating five paediatric falls assessment instruments and injury risk indicators: an ambispective study in a tertiary care setting. J Nurs Manag. Jul 2010;18(5):531-541.

63. Oliver D, Daly F, Martin FC, McMurdo ME. Risk factors and risk assessment tools for falls in hospital in-patients: a systematic review. Age Ageing. Mar 2004;33(2):122-130.

64. Harrington L, Luquire R, Vish N, et al. Meta-analysis of fall-risk tools in hospitalized adults. J Nurs Adm. Nov 2010;40(11):483-488.

65. Scott V. Multifactorial and functional mobility assessment tools for fall risk among older adults in community, home-support, long-term and acute care settings. Age Ageing. 2007;36:130-139.
